# Combinatorial Treatment of Human Cardiac Engineered Tissues With Biomimetic Cues Induces Functional Maturation as Revealed by Optical Mapping of Action Potentials and Calcium Transients

**DOI:** 10.3389/fphys.2020.00165

**Published:** 2020-03-12

**Authors:** Andy On-Tik Wong, Nicodemus Wong, Lin Geng, Maggie Zi-ying Chow, Eugene K. Lee, Hongkai Wu, Michelle Khine, Chi-Wing Kong, Kevin D. Costa, Wendy Keung, Yiu-Fai Cheung, Ronald A. Li

**Affiliations:** ^1^Department of Paediatrics and Adolescent Medicine, Li Ka Shing Faculty of Medicine, The University of Hong Kong, Pok Fu Lam, Hong Kong; ^2^Stem Cell and Regenerative Medicine Consortium, Li Ka Shing Faculty of Medicine, The University of Hong Kong, Pok Fu Lam, Hong Kong; ^3^Dr. Li Dak-Sum Research Centre, The University of Hong Kong – Karolinska Institutet Collaboration in Regenerative Medicine, The University of Hong Kong, Pok Fu Lam, Hong Kong; ^4^Department of Biomedical Engineering, University of California, Irvine, Irvine, CA, United States; ^5^Division of Biomedical Engineering, The Hong Kong University of Science and Technology, Kowloon, Hong Kong; ^6^Icahn School of Medicine at Mount Sinai, Manhattan, NY, United States; ^7^Ming-Wai Lau Centre for Reparative Medicine, Karolinska Institutet, Stockholm, Sweden

**Keywords:** maturation, tissue engineering, action potential, calcium handling, triiodothyronine, electrical conditioning

## Abstract

Although biomimetic stimuli, such as microgroove-induced alignment (μ), triiodothyronine (T3) induction, and electrical conditioning (EC), have been reported to promote maturation of human pluripotent stem cell-derived cardiomyocytes (hPSC-CMs), a systematic examination of their combinatorial effects on engineered cardiac tissue constructs and the underlying molecular pathways has not been reported. Herein, human embryonic stem cell-derived ventricular cardiomyocytes (hESC-VCMs) were used to generate a micro-patterned human ventricular cardiac anisotropic sheets (hvCAS) for studying the physiological effects of combinatorial treatments by a range of functional, calcium (Ca^2+^)-handling, and molecular analyses. High-resolution optical mapping showed that combined μ-T3-EC treatment of hvCAS increased the conduction velocity, anisotropic ratio, and proportion of mature quiescent-yet-excitable preparations by 2. 3-, 1. 8-, and 5-fold (>70%), respectively. Such electrophysiological changes could be attributed to an increase in inward sodium current density and a decrease in funny current densities, which is consistent with the observed up- and downregulated *SCN1B* and *HCN2/4* transcripts, respectively. Furthermore, Ca^2+^-handling transcripts encoding for phospholamban (PLN) and sarco/endoplasmic reticulum Ca^2+^-ATPase (SERCA) were upregulated, and this led to faster upstroke and decay kinetics of Ca^2+^-transients. RNA-sequencing and pathway mapping of T3-EC-treated hvCAS revealed that the TGF-β signaling was downregulated; the TGF-β receptor agonist and antagonist TGF-β1 and SB431542 partially reversed T3-EC induced quiescence and reduced spontaneous contractions, respectively. Taken together, we concluded that topographical cues alone primed cardiac tissue constructs for augmented electrophysiological and calcium handling by T3-EC. Not only do these studies improve our understanding of hPSC-CM biology, but the orchestration of these pro-maturational factors also improves the use of engineered cardiac tissues for *in vitro* drug screening and disease modeling.

## Introduction

The self-renewing property of human pluripotent stem cells (hPSC), including induced pluripotent stem cells (iPSC) and embryonic stem cells (ESC), offers a potentially unlimited supply of cardiomyocytes (CMs). Over the past decade or so, significant progress has been made in ventricular (V) specification to obtain high-purity VCMs (Ebert et al., 2012; Spater et al., 2014; Weng et al., 2014) for cardiac tissue engineering (Tallawi et al., 2015; Kolanowski et al., 2017; Wong et al., 2019). However, human pluripotent stem cell-derived cardiomyocytes (hPSC-CMs) possess immature fetal-like properties (Lieu et al., 2013; Robertson et al., 2013; Poon et al., 2015; Keung et al., 2016; Kadota et al., 2017; Ronaldson-Bouchard et al., 2019). Electrophysiologically, hPSC-CMs spontaneously fire action potentials (AP) with slower kinetics, starkly contrasting their quiescent-yet-excitable adult counterparts (Lieu et al., 2013). When assembled as monolayers, conduction is non-anisotropic and prone to arrhythmias unless the constituent cells are aligned (Chen et al., 2011; Luna et al., 2011; Wang et al., 2013; Shum et al., 2017). Furthermore, immature calcium handling properties (Liu et al., 2007, 2009; Li et al., 2013; Chen et al., 2015) contribute to weaker contractile functions (Ruan et al., 2016).

During the natural development and maturation of the cardiovascular system, a combination of specific stimuli is involved in a temporal and dynamic fashion (Peters et al., 1994; Robertson et al., 2013). Indeed, a number of non-cell autonomous factors have been investigated for their effects *in vitro* maturation of hPSC-CMs (Lieu et al., 2013; Yang et al., 2014a; Poon et al., 2015; Tzatzalos et al., 2016; Smith et al., 2017). For instance, triiodothyronine (T3), the active form of the thyroid hormone in humans, is crucial for normal cardiac development (Klein and Danzi, 2007) and promotes the expression of a wide range of calcium handling and contractile proteins in murine and human CMs (Danzi and Klein, 2002; Lee et al., 2010; Yang et al., 2014b). Electrical conditioning (EC), including chronic electrical field pacing and stimulation-induced active contraction, upregulates important ion channel and calcium handling transcripts, thereby improving the structural alignment and promoting contractility of hPSC-derived cardiac tissue constructs (Lieu et al., 2013; Ruan et al., 2016; Ronaldson-Bouchard et al., 2019). Although these different biological and environmental cues were identified (Robertson et al., 2013; Yang et al., 2014a; Denning et al., 2016), a combinatorial approach to synergistically promote electrophysiological and contractile maturation of hPSC-CMs has not been developed. Here, using the engineered cardiac construct human ventricular cardiac anisotropic sheets (hvCAS), we examined the electrophysiological consequences of microgroove-induced alignment, T3 and EC treatments, and this was followed by an investigation of the underlying molecular changes and pathways.

## Materials and Methods

### Cell Maintenance and Cardiac Specific Differentiation

The human embryonic stem cell line HES-2 (NIH code ES02, WiCell, United States) was maintained in mTeSR culture medium (Stem Cell Technologies) with 5% CO_2_ at 37°C. HES-2 was differentiated into ventricular CMs by using activin A, BMP-4, and IWR1, as reported previously (Weng et al., 2014; Shum et al., 2017). The differentiation cultures were maintained in complete StemPro 34 medium in a hypoxic condition (5% O_2_) for the first 8 days and then transferred into a normoxic incubator with 5% CO_2_. The culture media were replenished every 3–4 days until they were used for experiments.

### Fabrication of Substrates, Formation of hvCAS, and Combinatorial T3-EC Treatment

The microgrooved (μ) substrates 8, 10, and 15 μ with discrete dimensions [8 × 5 × 5, 10 × 5 × 5, and 15 × 5 × 5, Width (W) × Depth (D) × Ridge (R) in μm, respectively] were fabricated as previously reported (Shum et al., 2017). The surface of the substrates was activated by 8-min ultraviolet-ozone treatment (Jetlight UVO) followed by overnight coating of Matrigel at 4°C. Quality control of the differentiated human embryonic stem cell-derived cardiomyocytes (hESC-VCMs) was done by staining the cells with an anti-cardiac troponin T (cTnT) antibody (ab8295, Abcam) followed by flow cytometry. Only the batches with more than 65% cTnT-positive cells were used. Cardiospheres were dissociated with 0.025% trypsin-EDTA at 37°C for 15–20 min at day 15 post-differentiation. The cells were passed through a 40 μm Ø cell strainer to ensure complete dissociation. Cells were seeded onto the Matrigel-coated substrate (15 mm in diameter) with a density of 0.25 M cell/cm^2^. The hvCAS formed were maintained in Dulbecco’s modified Eagle medium (DMEM) with 10% FBS for 2 days, and they were then switched to the RPMI 1640 medium with B27 supplement (Gibco by Life Technologies) with the culture medium being refreshed every 2 days. For combinatorial T3-EC treatment, hvCAS were treated with additional 100 nM T3 from day 0 (day of hvCAS formation) to day 8. Non-T3-treated groups were subjected to basal T3 concentration at 2.6 nM (Chen et al., 2008). A stepwise EC protocol was adopted from day 3–8 (day 3: 0.2 Hz, day 4: 0.5 Hz, day 5–8: 1 Hz). The stimulation voltage and impulse duration were kept at 2.5 V/cm and 5 ms, respectively, throughout the treatment period. Where necessary, 10 μM SB431542 (TOCRIS) and 20 ng/ml recombinant human TGF-β1 protein (R&D System) were added to Flat untreated and 10 μ T3-EC groups, respectively, throughout the whole culture period. The properties of the hvCAS were studied at day 8 after formation.

### Immunofluorescent Imaging

Samples were fixed with 4% paraformaldehyde in phosphate-buffered saline (PBS) overnight at 4°C. After rinsing with PBS, they were permeabilized in PBS containing 1% Triton X-100 and subsequently blocked in 1% bovine serum albumin (BSA). Primary antibodies (Abcam) were diluted in PBS with 1% BSA at 1:200 and incubated at room temperature for 2 h. Alexa Fluor (AF) 488-conjugated goat anti-rabbit IgG or AF555 anti-mouse IgG (Invitrogen) were used as secondary antibodies at 1:1000 dilution and incubated for 1 h at room temperature. Prolong Gold mounting medium with DAPI (Invitrogen) was used to mount the samples under coverslips. The stained samples were then imaged on LSM Carl Zeiss 700 (Carl Zeiss).

### Measurement of hvCAS Spontaneous Contraction

Spontaneous contraction of hvCAS from each treatment group was observed under a bright field microscope on day 8. For each examined hvCAS, three different regions were observed randomly under 10× magnification. If no spontaneous contraction was observed for 15s, the region would be marked as a “quiescent region.” hvCAS were only defined as quiescent when each of the three observed regions were considered a “quiescent region.”

### Action Potential and Calcium Transient Optical Mapping

Human ventricular cardiac anisotropic sheet preparations were stained with 10 μM di-8-ANEPPS (Molecular Probes) with 0.04% Pluronic F-127 (Life Technologies) in serum-free DMEM/F12 for 30 min at 37°C. To eliminate motion artifacts, the samples were loaded with 50 μM blebbistatin (Sigma-Aldrich) for 15 min at room temperature before optical mapping. Tyrode’s solution, containing (in mM) 140 NaCl, 5 KCl, 1 MgCl_2_, 1 CaCl_2_, 10 D-glucose, and 10 4-(2-hydroxyethyl)-1-piperazineethanesulfonic acid (HEPES) at pH 7.4, was used in blebbistatin loading and during optical mapping experiments. The temperature of the samples was kept at 37°C. A high-resolution MiCAM Ultima optical mapping system (SciMedia) with a 1× lens setup was used to capture the AP signal and conduction velocities of the samples in a 1 cm × 1 cm region of interest. hvCAS was triggered by a unipolar point-stimulation electrode (Harvard Apparatus) and a programmable Master8 stimulator (AMPI). The recording frame rate was 5 ms. Data were analyzed by BVAna software (SciMedia). For calcium transient (CaT) optical mapping, samples were loaded with the calcium sensitive dye X-Rhod-1 AM (2 μM) (Life Technologies) for 30 min at 37°C followed by blebbistatin loading and the aforementioned steps.

### Redissociation of hvCAS and Electrophysiology Studies

The hvCAS was redissociated with 0.05% trypsin-EDTA solution for 3 min at 37°C at day 8. The cells were then seeded onto Matrigel-coated glass coverslip and allowed to recover for 48 h. Electrophysiological experiments were performed using whole-cell patch-clamp technique with EPC-10 amplifier and Pulse software (Heka Elektronik). For AP and *I*_*f*_ recording at 37°C, the internal solution contained (in mM): 110 K^+^ aspartate, 20 KCl, 1 MgCl_2_, 0.1 Na-GTP, 5 Mg-ATP, 5 Na_2_-phospocreatine, 1 EGTA, and 10 HEPES, and the pH was adjusted to 7.3 with KOH. The external Tyrode’s bath solution consisted of (mM): 140 NaCl, 5 KCl, 1 CaCl_2_, 1 MgCl_2_, 10 glucose, and 10 HEPES, and the pH was adjusted to 7.4 with NaOH. For *I*_*Na*_ recording at room temperature, internal solution contained (in mM): 135 CsCl, 10 NaCl, 2 CaCl_2_, 5 EGTA, 10 HEPES, and 5 MgATP, and the pH was adjusted to 7.2 with CsOH. The external solution contained (in mM): 25 NaCl, 135 CsCl, 1.8 CaCl_2_, 1 MgCl_2_, 10 Glucose, 10 HEPES, and 0.001 Nifedipine, and the pH was adjusted to 7.4 with CsOH. All reagents used were of analytical grade (Sigma-Aldrich). Curve fitting and plotting were performed using Prism 5 (GraphPad Software Inc.).

### Quantitative Real-Time PCR

From the samples, RNA was extracted with Trizol (Invitrogen) following the manufacturer’s protocol. A QuantiTect Reverse Transcription Kit (Qiagen) was used to prepare cDNA. For quantifying gene expressions, a StepOnePlus^TM^ Real-Time PCR System (Applied Biosystems) was used. PCR amplifications were carried out in 96-well optical plates with 20 μL reaction volume consisting of 100 ng of cDNA template, 4 ρmol of forward and reverse primers, and 1X KAPA SYBR FAST qPCR Master Mix (KAPA Biosystems). The reactions were incubated at 95°C for 3 min followed by 40–50 cycles of 95°C for 3 s and 60°C for 20 s. Primer sequences are available upon request.

### RNA Sequencing and Pathway Analysis

RNA samples of the hvCAS were harvested and extracted by Trizol as mentioned before. DNA libraries were prepared by KAPA Stranded mRNA-Seq Kit (Kapa Biosystems). HiSeq PE Cluster Kit v4 with cbot and HiSeq SBS Kit v4 (Illumina) was used for Pair-End 101bp sequencing by HiSeq 1500 (Illumina). Manufacturers’ protocols were followed. Sequencing reads were first filtered for the adapter sequence and low-quality sequence, and only reads with a read length ≥40 bp were retained. The rRNA-filtered raw reads showed alignment (human reference genome GRCh37 using TopHat) and transcriptome assembly (Cufflink). For each collection of gene sets, the gene sets with FDR-corrected *p*-values less than 0.20 were deemed significantly varied (enriched or depleted) ones.

## Statistical Analysis

Data were presented as mean ± standard error of the mean (SEM). Statistical comparisons were evaluated by an unpaired *t*-test for electrophysiology, a chi-square test for quiescence study, and a one/two-way ANOVA test followed by Dunnett’s/Tukey’s multiple comparison for other studies. The accepted level of significance for the tests was *p*-value <0.05.

## Results

### Combinatorial Effect of Microgroove, Triiodothyronine, and Electrical Conditioning on hvCAS

**FIGURE 1:**
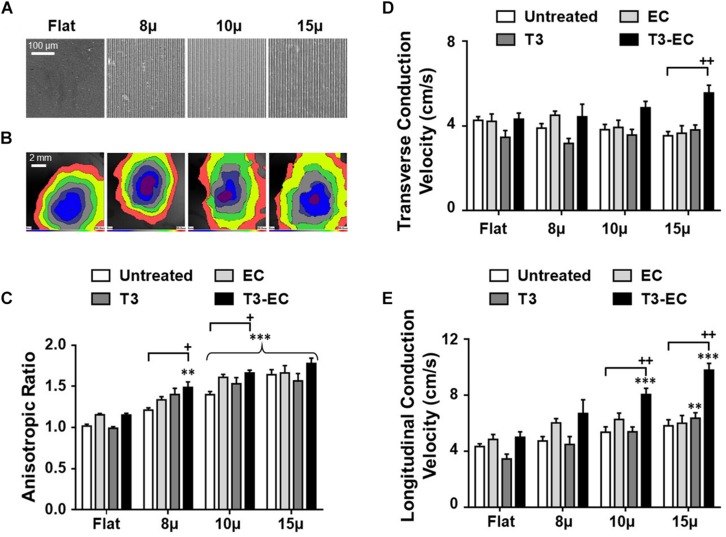
Microgroove-induced alignment (μ), triiodothyronine (T3), and electrical conditioning (EC) promote conduction anisotropy synergistically by increasing longitudinal conduction velocity (LCV). **(A)** Microscopic view of flat and microgrooved substrates with defined dimensions. **(B)** Isochronal map showing the induced anisotropy in AP conduction in microgrooved hvCAS when compared to that of flat control. **(C)** Anisotropic ratio (AR), **(D)** transverse conduction velocity (TCV), and **(E)** longitudinal conduction velocity (LCV) of hvCAS with different substrates and different treatment conditions. Data was generated by seven batches of independent differentiations; *n* = 5–9 for each group. **(C–E)** Two-way ANOVA test followed by Turkey multiple comparison test. ^∗^compared to flat control with the same treatment; +, compared to untreated condition of the same substrate. ^+^ indicates *p* < 0.05, ^∗∗^ or ^++^ indicate *p* < 0.01, and ^∗∗∗^ indicates *p* < 0.001.

The microgrooved (μ) substrates 8, 10, and 15 μ with discrete dimensions (8 × 5 × 5, 10 × 5 × 5, and 15 × 5 × 5, Width (W) × Depth (D) × Ridge (R) in μm, respectively) were fabricated as previously reported (Shum et al., 2017; Figure 1A). Differentiated hESC-VCM were cultured on flat control (flat) and microgrooved substrates for 8 days to form hvCAS. The percentage of cells with nuclei aligned along the microgroove direction significantly increased on 8, 10, and 15 μ by 54.3% (*p* = 0.07), 58.3% (*p* < 0.05), and 105.2% (*p* < 0.001), respectively, when compared to the control (flat). The anisotropic ratio (AR) for control (flat) was 1.0, which was consistent with the radial conduction of AP from the point of origin, as shown in the isochronal map. By contrast, the isochronal maps of hvCAS displayed anisotropic propagation of AP (Figure 1B) with the AR increasing with the 10 and 15 μ substrates (*p* < 0.05). Representative immunostaining images of α-actinin in flat, 8, 10, and 15 μ groups of hvCAS were used to demonstrate the change in tissue morphology (Supplementary Figure 1).

We next investigated the effects of T3, EC, and combined T3-EC treatments. While all of T3, EC, and combined T3-EC treatments further promoted AR for each of 8, 10, and 15 μ hvCAS (except for flat controls, which showed no statistically significant effect), the T3-EC groups consistently displayed the largest increases (Figure 1C). Indeed, T3-EC-treated 15 μ hvCAS also showed the highest transverse and longitudinal conduction velocities (Figures 1D,E). However, T3-EC had no effect on the nuclear orientation or circularity of all groups (data not shown). Collectively, our results suggested that topographical cues alone sufficed to induce cell alignment and further primed the hvCAS for augmented pro-maturational effect on AR and CVs by T3, EC, and T3-EC.

### Electrophysiological Basis of the Effect of T3-EC Treatment of hvCAS

**FIGURE 2:**
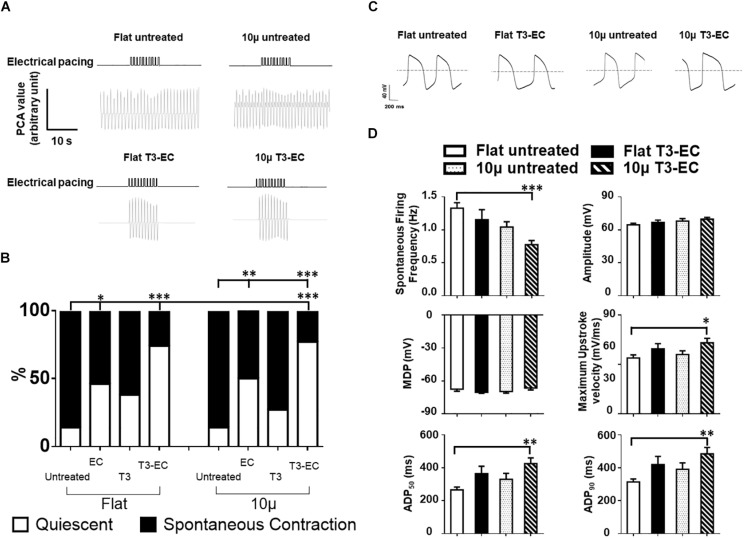
Combined T3-EC treatment promotes the formation of quiescent but excitable hvCAS with a more matured electrophysiology. **(A)** Representative tracings of optical flow analysis showing the quiescent but excitable properties of hvCAS after T3-EC treatment. **(B)** Combined T3-EC treatment significantly reduces spontaneous contraction in hvCAS (14 batches of independent differentiations; sample size: flat untreated, flat T3-EC, 10 μ untreated, and 10 μ T3-EC = 31–36, flat EC, flat T3, 10 μ EC, and 10 μ T3 = 11–13). **(C)** Representative spontaneous AP tracing of single hESC-VCMs redissociated from hvCAS by whole-cell patch clamping. **(D)** Spontaneous AP parameters (four batches of independent differentiations; *n* = 15–24 for each group). **(A)** Chi-square test between two groups. **(D)** One-way ANOVA test, followed by Dunnett multiple comparison test to flat untreated control. ^∗^*p* < 0.05, ^∗∗^*p* < 0.01, and ^∗∗∗^*p* < 0.001.

Since automaticity is considered an immature trait of hPSC-CMs, we examined the percentage (%) of quiescent hvCAS in our preparations. Upon EC treatment, the quiescent population of flat and 10 μ hvCAS increased from 14.7 and 14.3% to 46.2 and 50.0%, respectively (Figure 2B); T3 treatment led to similar increases (38.5% in Flat T3 and 27.8% in 10 μ T3). Interestingly, combined T3-EC treatment of flat and 10 μ hvCAS further increased to 74.2 and 77.1%, respectively (Figure 2B). Of note, all quiescent hvCAS preparations, treated or not, remained excitable upon field stimulation, thus confirming their electrical integrity (Figure 2A). However, unlike AR and CV, the effects of EC, T3, and T3-EC on automaticity did not seem to depend on microgroove-induced alignment.

**FIGURE 3:**
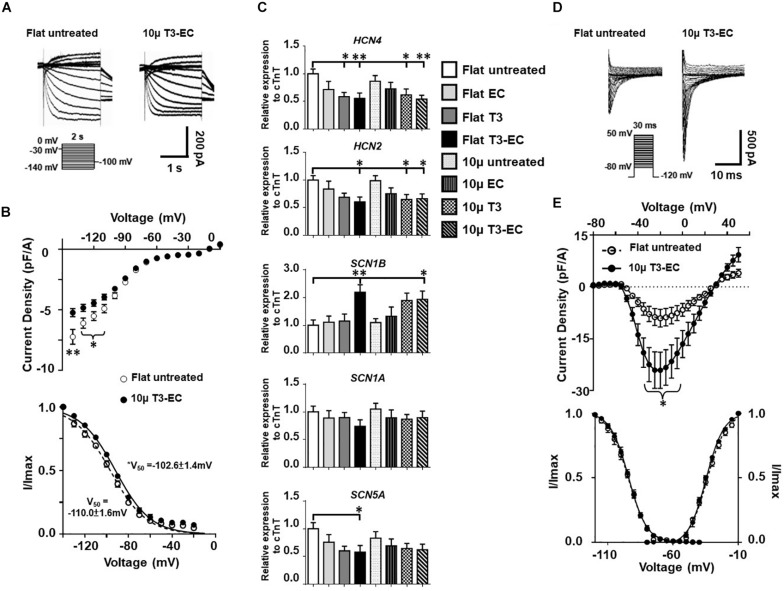
M-T3-EC decreases funny current by downregulating HCN2/4 expression and increases inward sodium current by upregulating SCN1B expression in hvCAS. **(A)** Representative *I*_*f*_ traces in the presence of 1mM Ba^2+^ of flat untreated and 10 μ T3-EC hvCAS. **(B)** The steady-state I–V and steady-state activation relations of *I*_*f*_ from flat untreated and 10 μ T3-EC groups (three batches of independent differentiations; *n* = 16–21). **(C)** mRNA expression level of HCN4, HCN2, SCN1B, SCN1A, and SCN5A (11 batches of independent differentiations, *n* = 11). **(D)** Representative *I*_*Na*_ traces of flat untreated and 10 μ T3-EC hvCAS. **(E)** The peak *I*_*Na*_ density I–V plots, steady-state activation (G/Gmax), and inactivation (I/Imax) relationships of flat untreated and 10 μ T3-EC groups (peak *I*_*Na*_ density I–V plots and steady-state activation: three batches of independent differentiations, *n* = 9–11; inactivation: two batches, *n* = 5–7). **(B,E)** Two-tailed unpaired *t*-test. **(C)** One-way ANOVA test followed by Dunnett multiple comparison test to flat untreated control. ^∗^*p* < 0.05 and ^∗∗^*p* < 0.01.

To obtain mechanistic insights into the underlying ionic basis, single hESC-VCMs were re-dissociated from untreated and T3-EC-treated flat and 10 μ hvCAS for whole-cell patch-clamp recordings. Representative tracings of spontaneous AP-firing are shown in Figure 2C. T3-EC-treated 10 μ hvCAS displayed significantly slower AP-firing with a faster maximum upstroke velocity as well as prolonged APD_50_ and APD_90_ during either spontaneous firing or current-clamped pacing conditions compared to those of untreated and treated flat as well as untreated 10 μ (Figure 2D). However, MDPs were not different among the groups. Similar results were observed in the 1Hz paced condition (Supplementary Figure 2). Given its importance in automaticity (Azene et al., 2005; Tse et al., 2006; Xue et al., 2007; Lieu et al., 2008; Sun et al., 2017), we next measured the funny current (*I*_*f*_) in hESC-VCMs isolated from hvCAS. Representative tracings were shown in Figure 3A. Voltage-clamp recordings showed a significant reduction of *I*_*f*_ current density in the T3-EC-treated 10 μ group at hyperpolarizing potentials (from −110 to −140 mV; Figure 3B). Paradoxically, a significant positive shift of the V1/2 was also observed. Consistent with a decrease in the current density, *HCN2* and *HCN4* transcripts that underlie *I*_*f*_ (Kim et al., 2010) were downregulated (Figure 3C).

Since elevated maximum upstroke velocity was detected in T3-EC-treated 10 μ hvCAS, we next studied the inward sodium current (*I*_*Na*_). Figures 3D,E showed a significantly increased *I*_*Na*_ of the T3-EC-treated 10 μ group, although the steady-state activation and inactivation properties of *I*_*Na*_ were not altered (Figure 3E). Such an increase in *I*_*Na*_ was likely due to the increased expression of SCN1B, the beta-unit, rather than SCN1A or SCN5A, which were the pore-forming alpha subunit, as indicated by the expression levels of their transcripts normalized to that of cTnT (Figure 3C). Gene expression in hvCAS normalized to housekeeping gene GAPDH showed similar results (Supplementary Figure 3).

### T3-EC Improved Calcium Handling Properties

**FIGURE 4:**
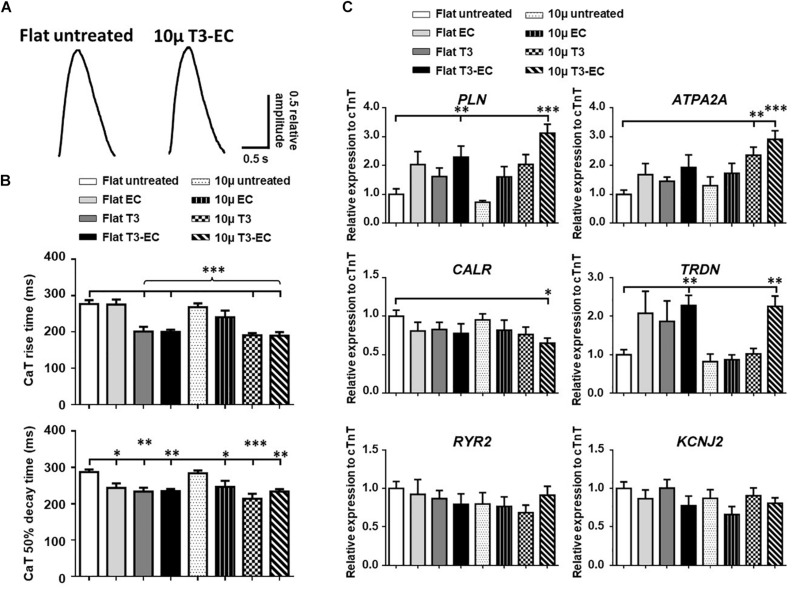
T3-EC improves calcium handling properties in hvCAS. **(A)** Representative tracings by calcium transient (CaT) optical mapping hvCAS with different treatments. **(B)** CaT rise time and 50% decay time (CaT_50_) measured by calcium transient optical imaging (three batches of independent differentiations; *n* = 5–6 for each group). **(C)** Changes in mRNA expression levels of calcium handling genes (11 batches of independent differentiations). **(B,C)** One-way ANOVA test followed by Dunnett multiple comparison test to flat untreated group. ^∗^*p* < 0.05, ^∗∗^*p* < 0.01, and ^∗∗∗^*p* < 0.001.

Representative Ca^2+^ transient tracings of hvCAS as optically mapped are shown in Figure 4A. The rise time and time to 50% decay for T3-, EC-, and T3-EC-treated 10 μ hvCAS were reduced when compared to the untreated controls (Figure 4B). Consistently, the Ca^2+^ handling genes *PLN* and *ATP2A2*, which encode for phospholamban (PLN) and sarco/endoplasmic reticulum Ca^2+^-ATPase (SERCA), respectively, were upregulated by up to 3-fold in the T3-EC-treated 10 μ group. TRDN, which encodes for the gene triadin, was also upregulated by 2.5-fold in the T3-EC-treated flat as well as 10 μ groups (Figure 4C).

### Combinatorial Treatment Commonly Downregulated the TGF-β Signaling Pathway

**FIGURE 5:**
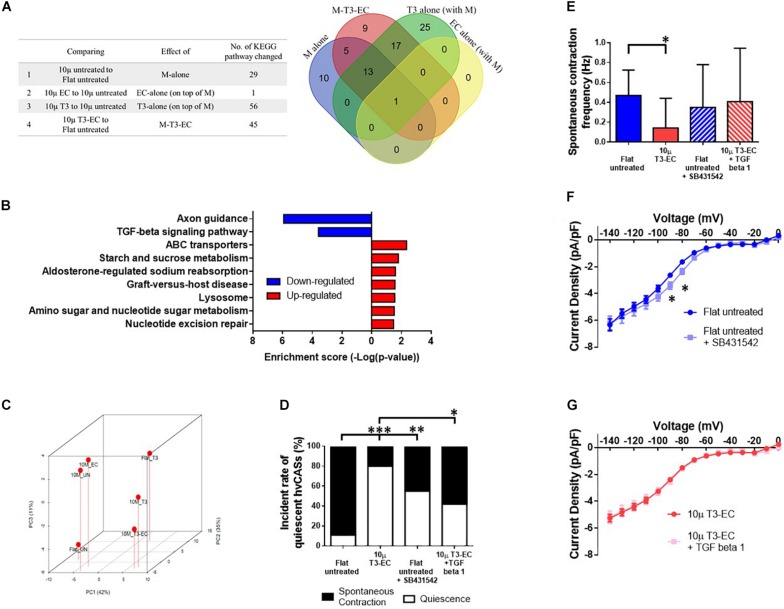
TGF-β signaling pathway downregulation is associated with T3-EC-induced quiescence. **(A)** The list and the corresponding Venn diagram showing the significant changed pathways of different conditions and combinatorial treatment by the KEGG pathway database. **(B)** The enrichment score of the KEGG pathways significantly changed. **(C)** The principal component analysis (PCA) showing the relative relationship of TGF-β signaling pathway expression level of each groups. The relationships of SB431542 and/or TGF-β1 in flat untreated and 10 μ T3-EC hvCAS, respectively. **(D)** The incident rate of quiescent hvCAS. **(E)** The spontaneous contraction frequency of hvCAS (three batches of independent differentiations, *n* = 14–18 for each group). **(F,G)** The steady-state I–V relationships of *I*_*f*_ from flat untreated and 10 μ T3-EC with SB431542 and TGF-β1, respectively (two–three batches of independent differentiations, *n* = 10–16). **(D)** Chi-square test. **(E)** One-way ANOVA test, followed by Dunnett multiple comparison test to flat untreated control. **(F,G)** Two-tailed unpaired *t*-test. ^∗^*p* < 0.05, ^∗∗^*p* < 0.01, and ^∗∗∗^*p* < 0.001.

To obtain insight into the molecular pathways that underlie the observed pro-maturation effects of the various conditions tested, we performed RNA-sequencing of untreated flat, un-, T3-, EC-, and T3-EC-treated 10 μ hvCAS followed by systematic bioinformatics analyses. By comparing different conditions and mapping the differentially expressed genes onto the Kyoto Encyclopedia of Genes and Genomes (KEGG) pathway database^1^, the number of pathways with significant changes in gene expression for each treatment group was plotted in the Venn diagram shown in Figure 5A. The pathway that was commonly enriched by μ-/EC-/T3-alone and μ-T3-EC-treated hvCAS was the ribosome pathway. However, this pathway was upregulated in the μ-alone condition but downregulated in the EC-/T3-alone and μ-T3-EC conditions. On the other hand, the transforming growth factor beta (TGF-β) signaling pathway was identified as one of the three most significantly changed signaling pathways in μ-T3-EC-treated hvCAS but was not found in the μ-/EC-/T3- alone condition together with axon guidance and ATP-binding cassette (ABC) transporters pathways (Figure 5B). Figure 5C shows the PCA plot of the TGF-β signaling pathway. The expression of *TGFB1* (TGF-β1 protein), *TGFBR1* (Transforming Growth Factor Beta Receptor 1), and the *SMAD2/3* (the downstream signal transducers of TGF-β1) were decreased in the μ-T3-EC-treated hvCAS when compared to control (data not shown). As a proof-of-concept experiment to test the role of downregulating the TGF-β signaling pathway in μ-T3-EC-induced maturation, recombinant human TGF-β1 protein and the TGFBR1 antagonist SB431542 were added to the 10 μ T3-EC and untreated flat hvCAS, respectively. SB431542 significantly increased the % of quiescent hvCAS to 55.6% when compared to untreated; by contrast, TGF-β1 reversed the effect of T3-EC induced on reduced automaticity (% of quiescent hvCAS decreased to 42.9% from 80.0%) (Figure 5D). Similarly, the spontaneous contraction frequency was significantly reduced in 10 μ T3-EC compared to the untreated control group, and such statistical difference was abolished after treating 10 μ T3-EC with TGF-β1 (Figure 5E). Interestingly, the *I*_*f*_ current density was right shifted after treating the Flat untreated group with SB431542 (Figure 5F). No change in *I*_*f*_ current density could be detected in 10 μ T3-EC with or without TGF-β1 treatment (Figure 5G).

## Discussion

The present study investigated the effect of topographical, hormonal, and electrical cues on the electrophysiological and Ca^2+^-handling properties of engineered hvCAS. Our results showed that μ-T3-EC induced a more mature phenotype with reduced automaticity, mature electrophysiology, and augmented Ca^2+^ handling in comparison with the individual treatments alone, highlighting the importance of combinatorial application of appropriate biomimetic stimuli in driven maturation. Mechanistically, topographical cues alone induced cell alignments, and further primed the hvCAS for augmented pro-maturational effects on AR and CVs by T3, EC, and T3-EC. Electrophysiologically, an increase in *I*_*Na*_ resulting from an upregulated beta- but not alpha-subunit, could underlie the hastened conduction, while the reduced automaticity as a sign of maturation could be attributed to the reduced *I*_*f*_ due to the downregulation of their molecular correlates *HCN2* and *HCN4*. As for Ca^2+^ handling, which is of central importance to excitation–contraction coupling, such related gene products as *PLN* and *SERCA* were upregulated, leading to improved calcium handling and subsequent contractility (Keung et al., 2016; Li et al., 2019). Other groups have reported similar findings where electrical pacing in hiPSC-CM tissue constructs promoted contractile force by ∼20-fold along with ∼2- and ∼1.5-fold increases in protein expression of SERCA and RYR, respectively, when compared to untreated controls (Ruan et al., 2016) or sensitivity toward isoproterenol, as indicated by calcium imaging (Ronaldson-Bouchard et al., 2019).

Another finding of this study is the identification of the downregulation of the TGF-β signaling pathway induced by T3-EC combinatorial treatment. While TGF-β has been shown in a number of studies to be involved in cardiac development and is essential for efficient differentiation of hPSCs to CMs (MacLellan et al., 1993; Camenisch et al., 2002; Watabe and Miyazono, 2009), elevated TGF-β signaling in the human heart is also associated with various pathological conditions, including hypertrophic cardiomyopathy (Dobaczewski et al., 2011). Chronic exposure to TGF-β1 increases RyR-mediated spontaneous Ca^2+^ oscillation (Neylon et al., 1994) and spontaneous beating frequency (Carrillo et al., 1998), while reducing *I*_*Na*_ and inward rectifier potassium current (*I*_*K*__1_) densities (Ramos-Mondragon et al., 2011) in isolated neonatal rat CMs. Our findings that a blockade of TGF-β1 receptors was associated with a more quiescent hvCAS is consistent with this notion. There was no significant change in *I*_*f*_ between the hESC-VCM treated with T3-EC and that with TGF-β1. As such, it remains unclear how TGF-β1 led to our observed effect of increased quiescence. However, several reports pointed to the alteration of different ion channel activities, including the inward rectifier potassium current (*I*_*K*__1_) (Lieu et al., 2013), *I*_*f*_ (Kolanowski et al., 2017) and the “calcium spark” from which calcium ions are released from the sarcomeric reticulum (SR) via overactive RyR (Li et al., 2013). Spontaneous contraction can be induced by spontaneous Ca^2+^ oscillation caused by the malfunction of calcium handling units, such as PLN (Lakatta et al., 2010; Li et al., 2013). A recent study in hiPSC-CM generated from a dilated cardiomyopathy (DCM) patient with PLN mutation showed an increase in the spontaneous beating rate, which could be reverted by genetic correction (Karakikes et al., 2015), and this suggested a link between T3-EC-induced *PLN* upregulation and the observed reduction in automaticity in our study. The expression of calreticulin (CALR) is low in a mature cardiomyocyte (Lynch et al., 2006), while triadin (TRDN) has been reported to assist the anchoring of calsequestrin (CASQ2) to the RyR, and the spontaneous calcium release could thus be stabilized and thus reduce the SR calcium leakage (Knollmann, 2009; Li et al., 2013). In M-T3-EC-treated hvCAS, the gene expression levels of *CALR* and *TRDN* was downregulated and upregulated, respectively, suggesting their role in the reduced automaticity was observed. RNA-seq and pathway mapping of T3-EC-treated hvCAS revealed that the TGF-β signaling was downregulated; the TGF-β receptor agonist and antagonist TGF-β1 and SB431542 partially reverted T3-EC induced quiescence and reduced spontaneous contractions, respectively.

Although it was demonstrated that the Ca^2+^-handling function was improved by the combinatorial treatment, one limitation of the current study was the lack of direct readout of contractility in these hPSC-CM engineered tissues. Proof-of-concept studies showed that the contractile function of 3D cardiac tissue constructs could be improved by EC in 2–4 weeks by various groups (Hirt et al., 2014; Ruan et al., 2016; Ronaldson-Bouchard et al., 2019), while, in the present study, it was observed that μ-T3-EC treatment could induce maturation in electrophysiology and calcium handling with ∼1 week. Further study is warranted to determine whether the combinatorial treatment regimen could exert a synergistic effect on hPSC-CM contractility by accelerating the electro-mechanical training effect of EC. It has been suggested that the immaturity of cardiac engineered tissue may render them insensitive to cardioactive drugs *in vitro* drug screening and cardiotoxicity testing. The combinatorial treatment described in the present study would greatly enhance the biofidelity of these engineered cardiac tissues by inducing maturation in 3D cardiac tissue models which are more physiological when compare to 2D tissue (Li et al., 2018; Lee et al., 2019; Ronaldson-Bouchard et al., 2019).

In sum, we conclude that topographical cues primed cardiac tissue constructs for augmented electrophysiological and contractile maturation by T3-EC. This study improves our understanding of hPSC-CM biology. Together with the development of various 3D cardiac organoid model, the capacity of matured engineered cardiac tissues for *in vitro* drug screening and disease modeling can be greatly enhanced.

## Data Availability Statement

The RNA-sequencing data generated in this study has been deposited to the BioProject database (accession no: PRJNA601167).

## Author Contributions

RL conceived the project. Y-FC and RL supervised the project. AW, C-WK, WK, and RL designed the experiments. AW, NW, LG, MC, and EL performed experiments. All authors contributed to data analysis and interpretation. AW, WK, Y-FC, and RL contributed to writing the manuscript.

## Conflict of Interest

MK, KC, and RL hold equity in Novoheart Holdings. Research conducted in this study could potentially affect the value of Novoheart. AW, NW, LG, MC, EL, HW, C-WK, WK, and Y-FC declared no competing interests for this work.

## Footnotes

1 http://www.genome.jp/kegg
