# Supplementary material for: Combinatorial Treatment of Human Cardiac Engineered Tissues With Biomimetic Cues Induces Functional Maturation as Revealed by Optical Mapping of Action Potentials and Calcium Transients
Source: Front Physiol. 2020 Mar 12;11:165. doi: 10.3389/fphys.2020.00165 (PMC7080659; doi:10.3389/fphys.2020.00165)
Supplement: Supplementary file 1 [file Data_Sheet_1.pdf]

Supplemental Figure 1. Immunostaining of hvCAS.

A) Representative immunostaining images of α-actinin in flat, 8μ, 10μ and 15μ groups of hvCAS. **B**) Representative immunostaining images of cTnT and connexin 43 in flat and10μ groups of hvCAS with or without T3-EC treatment.

Supplemental Figure 2. Combined T3-EC treatment promotes the formation of hvCAS with a more matured electrophysiology at paced condition.

A) Representative AP tracing of single hESC-VCMs redissociated from hvCAS by whole-cell patch clamping paced at 1Hz. **B**) AP parameters at 1Hz pacing frequency (4 batches of independent differentiations; n=15-24 for each group). Data are presented as mean ±SEM. Statistical analysis as determined by One-way ANOVA followed by post-hoc Dunnett's multiple comparison test. *$p<0.05$ vs flat untreated control.

Supplemental Figure 3. mRNA expression normalized to GAPDH of various genes in hvCAS.

mRNA expression levels of funny current channels, sodium channel units and calcium handling genes normalized to GAPDH in hvCAS were shown (11 batches of independent differentiations, n=11). Data are presented as mean ±SEM. Statistical analysis as determined by One-way ANOVA followed by post-hoc Dunnett's multiple comparison test. *$p<0.05$, **$p<0.01$, ***$p<0.001$ vs flat untreated control.
