# Supplementary figures and images for: Combinatorial Treatment of Human Cardiac Engineered Tissues With Biomimetic Cues Induces Functional Maturation as Revealed by Optical Mapping of Action Potentials and Calcium Transients
Source: Front Physiol. 2020 Mar 12;11:165. doi: 10.3389/fphys.2020.00165 (PMC7080659; doi:10.3389/fphys.2020.00165)

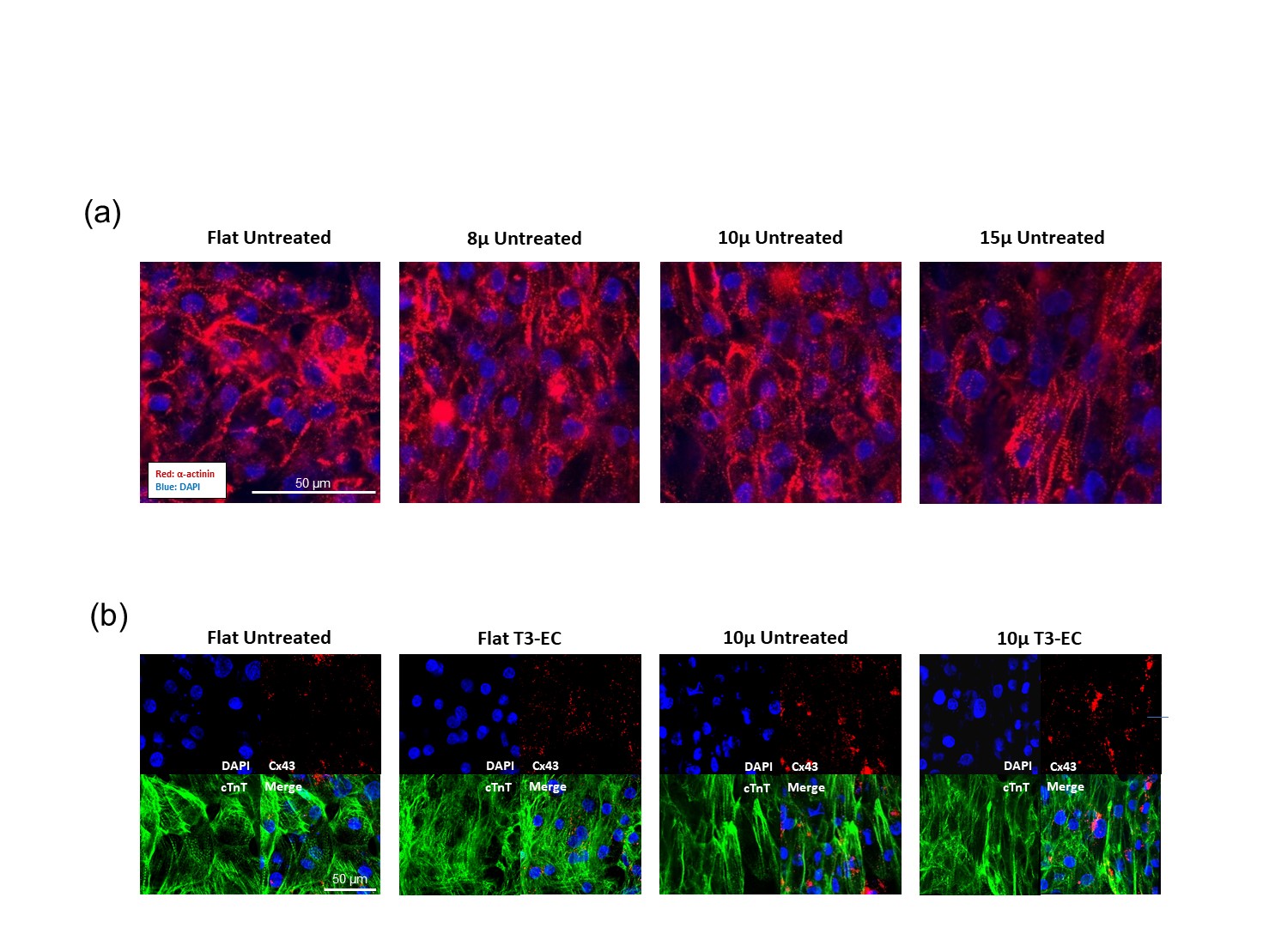

Supplement: Supplementary file 2 [file Image_1.JPEG]

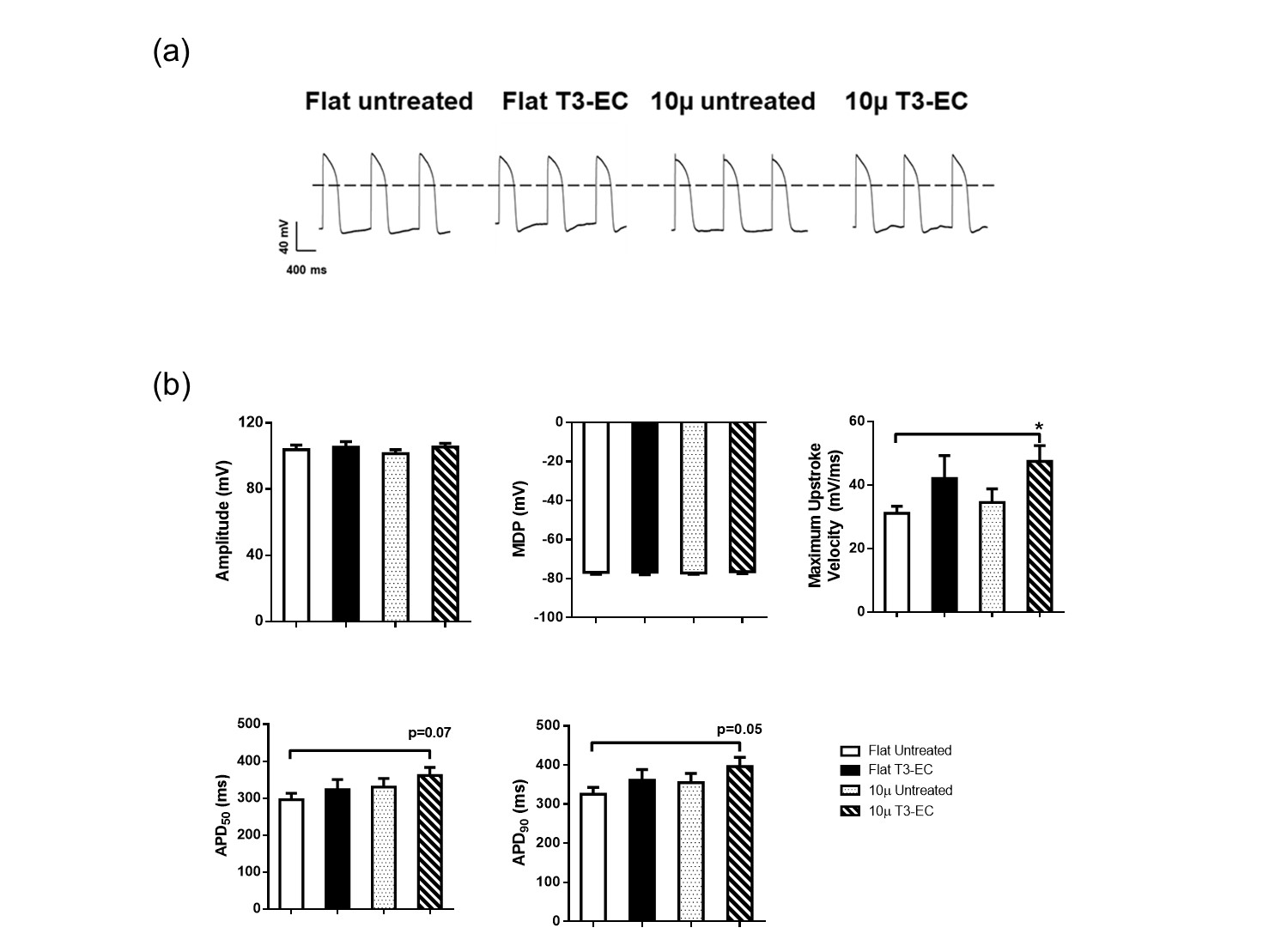

Supplement: Supplementary file 3 [file Image_2.JPEG]

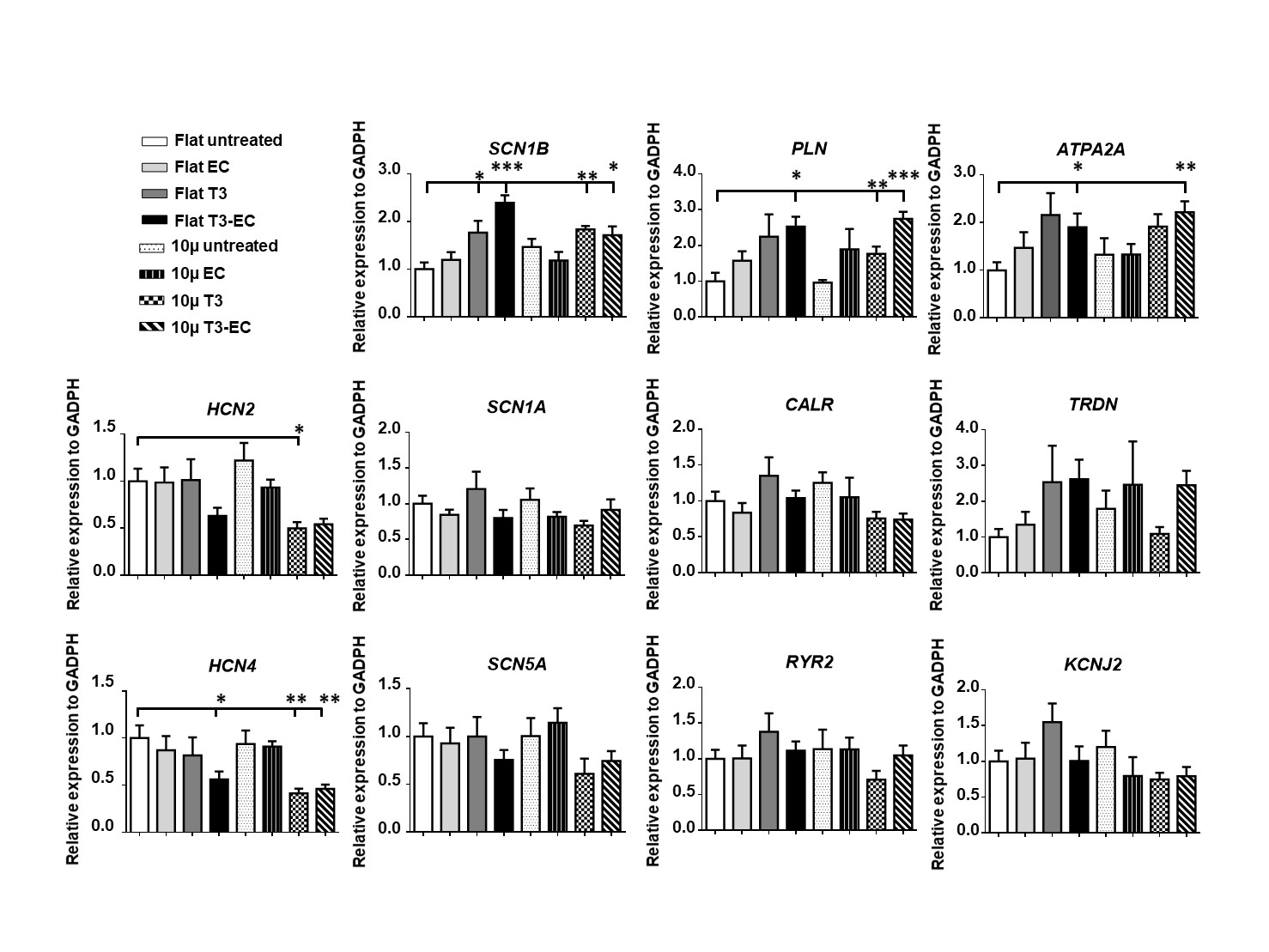

Supplement: Supplementary file 4 [file Image_3.JPEG]
